# AMP‐activated protein kinase α1 phosphorylates PHD2 to maintain systemic iron homeostasis

**DOI:** 10.1002/ctm2.854

**Published:** 2022-05-11

**Authors:** Cheng Wang, Wencheng Zhang, Wenjing Xu, Zhaoyu Liu, Kai Huang

**Affiliations:** ^1^ Clinic Center of Human Gene Research Union Hospital Tongji Medical College Huazhong University of Science and Technology Wuhan China; ^2^ Hubei Key Laboratory of Metabolic Abnormalities and Vascular Aging Tongji Medical College Huazhong University of Science and Technology Wuhan China; ^3^ Department of Rheumatology Union Hospital Tongji Medical College Huazhong University of Science and Technology Wuhan China; ^4^ Department of Cardiology Qilu Hospital Cheeloo College of Medicine Shandong University Jinan China; ^5^ Department of Cardiology Sun Yat‐sen Memorial Hospital Sun Yat‐sen University, Guangzhou China

**Keywords:** AMPK, hepcidin, HIF1α, iron, PHD2

## Abstract

**Background:**

Iron is essential for all mammalian life, and either a deficiency or excess of iron can cause diseases. AMP‐activated protein kinase (AMPK) is a critical regulator of metabolic homeostasis; however, it has not been established whether AMPK regulates iron metabolism.

**Methods:**

Iron, hepcidin and ferroportin levels were examined in mice with global and hepatocyte‐specific knockout of AMPKα1 and AMPKα2. Primary AMPKα1 or AMPKα2 deleted hepatocytes were isolated and cultured in hypoxia condition to explore PHD2, HIF and hydroxylated HIF1α levels. We performed immunoprecipitation, in vitro AMPK kinase assay and site‐direct mutant assay to detect phosphorylation sites of PHD2. We also obtained liver tissues from patients with anaemia of chronic disease undergoing surgery, AMPKα1 and hydroxylated HIF1α levels were measured by immunohistochemical analysis.

**Results:**

We found that mice with global deficiency of AMPKα1, but not AMPKα2, exhibited hypoferraemia as well as iron sequestration in the spleen and liver. Hepatocyte‐specific, but not myeloid‐specific, ablation of AMPKα1 also reduced serum iron levels in association with increased hepcidin and decreased ferroportin protein levels. Mechanistically, AMPKα1 directly phosphorylated prolyl hydroxylase domain‐containing (PHD)2 at serines 61 and 136, which suppressed PHD2‐dependent hydroxylation of hypoxia‐inducible factor (HIF)1α and subsequent regulation of hepatic hepcidin‐related iron signalling. Inhibition of PHD2 hydroxylation ameliorated abnormal iron metabolism in hepatic AMPKα1‐deficient mice. Furthermore, we found hepatic AMPKα/PHD2/HIFα/ hepcidin axes were highly clinically relevant to anaemia of chronic disease.

**Conclusion:**

In conclusion, these observations suggest that hepatic AMPKα1 has an essential role in maintaining iron homeostasis by PHD2‐dependent regulation of hepcidin, thus providing a potentially promising approach for the treatment of iron disturbances in chronic diseases.

## Graphical abstract

1. AMPKα1 mediates iron homeostasis via regulation of the hepcidin/ferroportin pathway
2. AMPKα1 regulated HIF1α hydroxylation and ubiquitination in a PHD2‐dependent manner.
3. AMPKα1 interacts and phosphorylates PHD2
4. Clinical relevance of AMPKα1/PHD2/hepcidin axis in systemic iron disturbances.

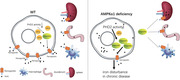

## 1 INTRODUCTION

Iron is a vital nutrient for nearly all living organisms because it is critical for oxygen delivery, various enzymatic and redox reactions for energy metabolism, endobiotic and xenobiotic metabolism and respiration.^1^, ^2^, ^3^ Iron deficiency leads to anaemia, growth arrest and cell death, whereas excessive iron can catalyse the formation of free radicals that damage DNA, lipid membranes and proteins via the Fenton reaction.^4^, ^5^, ^6^ The liver is crucially important for iron metabolism because hepatocytes produce hepcidin, a small antimicrobial peptide hormone that controls iron homeostasis by regulating the amount of ferroportin, a cellular iron exporter that transports absorbed, recycled or stored iron from tissues into the plasma.^7^, ^8^, ^9^ The binding of hepcidin to ferroportin leads to internalisation and degradation of ferroportin within lysosomes, which in turn results in decreased iron transport out of iron‐storing cells and reduced iron absorption by enterocytes.^2^, ^10^ Impaired hepcidin activity is associated with hereditary haemochromatosis caused by mutations in the genes encoding hepcidin, haemochromatosis protein, haemojuvelin and transferrin receptor 2.^11^, ^12^, ^13^ In contrast, high levels of circulating hepcidin are associated with macrophage iron loading, low iron levels in plasma and inhibited erythropoiesis, which is secondary to reduced transferrin‐bound iron.^13^ Reduced erythropoiesis leads to anaemia of chronic disease, a common condition in hospitalised patients.

In general, hepcidin can be upregulated by Smad proteins (homologues of the *Drosophila* protein, mothers against decapentaplegic (Mad) and the *Caenorhabditis elegans* protein Sma), which bind the bone morphogenetic protein (BMP)‐responsive element and transfer the ‘iron signal’, or by the interleukin (IL)‐6/signal transducer and activator of transcription (STAT)3 inflammatory signalling pathway.^14^, ^15^, ^16^ Given the links among oxygen transport, iron metabolism and erythropoiesis, potential hypoxia‐associated regulatory mechanisms might control hepcidin level in iron homeostasis.^17^ Hepcidin is suppressed by both hypoxia and anaemia.^18^ Furthermore, hypoxia‐inducible factor (HIF), a key element in the response to hypoxia, is post‐transcriptionally regulated by prolyl hydroxylase domain‐containing proteins (PHDs) and subsequently degraded through Von Hippel‐Lindau (VHL)‐related ubiquitin/proteasome pathway.^19^ Decreased tissue oxygenation suppresses the expression of hepcidin by inhibiting activity of PHD and VHL‐mediated HIF‐α factor degradation.^20^ These results, therefore, indicate that under hypoxic conditions, PHD/HIF is a critical link between hepcidin regulation and iron fluctuations. However, PHDs are not only regulated by hypoxia; other mechanisms such as iron itself, phosphorylation or tissue‐specific factors may also be involved. Thus, factors potentially regulating PHD activity and the downstream HIF–hepcidin pathway within the liver warrant further investigation.

AMP‐activated protein kinase (AMPK) is a master regulator of metabolic homeostasis and a cellular energy sensor. It is a heterotrimeric serine/threonine enzyme consisting of 1 catalytic (α1 or α2) subunit and 2 regulatory (β1 or β2 and γ1, γ2, or γ3) subunits.^21^, ^22^ The α subunit has two isoforms, α1 and α2, and contains a kinase domain. The liver expresses approximately equal amounts of α1 and α2.^23^ As its name suggests, AMPK is activated under conditions that elevate AMP/ATP ratios, including glucose deprivation, muscle contraction and hypoxia.^24^ AMPK is also activated by phosphorylation of the α subunit at Thr172 by at least 2 upstream kinases: calmodulin‐dependent protein kinase kinase (CaMKK) β and liver kinase (LK) B1.^25^ The role of AMPK in regulating energy homeostasis is related to its effects on glucose and lipid metabolism, as well as on mitochondrial biogenesis and function.^24^ However, it is currently unknown whether AMPK regulates iron metabolism.

This study aims to investigate the contributions of AMPKα in the regulation of iron homeostasis and hepcidin expression in the liver. Herein, we report that AMPKα1^–/–^ deletion, but not AMPKα2^–/–^ deletion, increases PHD2 activity and hepcidin expression, the latter of which inhibits iron absorption and recycling and finally leads to hypoferraemia and iron sequestration.

## 2 MATERIALS AND METHODS

### 2.1 Animals

All experimental procedures were approved by the Institutional Animal Care and Use Committee in Tongji Medical College of Huazhong University of Science and Technology. AMPKα1^–/–^ and AMPKα2^–/–^ mice were generated as described previously.^26^ Age‐matched WT littermates were used as controls. All the mice had the C57BL/6J genetic background. Liver‐specific AMPKα1 knockout (AMPKα1^fl/fl^Alb^+^) mice were generated by crossing AMPKα1^fl/fl^ mice with albumin‐cre mice. Myeloid‐specific AMPKα1 knockout (AMPKα1^fl/fl^Lyz^+/–^) mice were generated by crossing AMPKα1^flox/flox^ mice with lysozyme 2‐cre mice. The male mice were used in the experiments. For the DMOG (a 2‐oxoglutarate analogue that inhibits PHD2 hydroxylase activity) treatment, AMPKα1^fl/fl^Alb^–^ and AMPKα1^fl/fl^Alb^+^ mice were injected intraperitoneally once daily with 8 mg DMOG per mouse or its vehicle control. Four weeks later, mice were killed to analyse their serum and liver iron levels.

### 2.2 Serum iron measurements

Serum iron and unsaturated‐iron–binding capacity (UIBC) were detected by using the Iron/UIBC kit (Thermo Electron, Waltham, MA, USA). Transferrin saturation percentage was calculated as serum iron/TIBC (total iron‐binding capacity) × 100%. TIBC was calculated as the sum of the serum iron and UIBC.

### 2.3 Iron quantification

Non‐haeme iron quantification of liver tissue was performed using the bathophenanthroline assay, with modifications. Briefly, liver tissue was digested in 3 M HCl/10% trichloroacetic acid at 65°C for 72 h. The supernatants were collected after centrifugation at 10 000 × *g* for 5 min, followed by the addition of chromogen. The samples were then mixed by vortexing. Serial dilutions of a ferric iron standard (500 μg/dl; Sigma Diagnostics, Livonia, MI, USA) were used to construct a standard curve. Colour was allowed to develop and measured as absorbance at 535 nm.

### 2.4 Cell culture

HepG2 cells (HB‐8065; ATCC, Manassas, VA, USA) were grown in Minimum Essential Medium (Invitrogen, USA), supplemented with 10% foetal bovine serum (Invitrogen), 100 mg/ml streptomycin, 100 IU/ml penicillin and 2 mM L‐glutamine. Primary hepatocytes were isolated and cultured from mice as previously described.^27^ Briefly, after mice were anesthetised, their livers were perfused via the portal vein with freshly prepared perfusion medium (Invitrogen), followed by digestion buffer (0.33 mg of collagenase I/ml). The livers were subsequently dispersed with hepatocyte wash medium (Invitrogen), and then seeded onto fibronectin‐coated plates (Corning, USA). For hypoxia induction in vitro, cells were incubated in an airtight chamber in an atmosphere of 1% O_2_, 94% N_2_ and 5% CO_2_ for the time periods indicated.

### 2.5 Western blots and immunohistochemistry

Western blots and immunohistochemical staining were performed as described.^28^, ^29^, ^30^ Antibodies used were SLC40A1 (NBP1‐21502, Novus Biologicals, Centennial, CO, USA, 1:500), GAPDH (sc‐32233, Santa Cruz Biotechnology, Dallas, TX, USA, 1:2000), AMPKα1 (2795, Cell Signaling Technology, Danvers, MA, USA, 1:1000 and sc‐19128, Santa Cruz Biotechnology, 1:500), AMPK‐α2 (2757, Cell Signaling Technology, 1:1000 and sc‐19131, Santa Cruz Biotechnology, 1:1000), pAMPK (Thr172, 2535, Cell Signaling Technology, 1:1000), Smad1/5/8 (sc‐6031, Santa Cruz Biotechnology, 1:1000), pSmad1/5/8 (9511, Cell Signaling Technology, 1:1000), HIF1α (14179, Cell Signaling Technology, 1:1000), Hydroxy‐HIF1α (Pro564) (3434, Cell Signaling Technology, 1:1000), pSer/Thr (ab17464, Abcam, 1:1000 ), PHD2 (4835, Cell Signaling Technology, 1:1000), HA (3724, Cell Signaling Technology, 1:1000) and His (12698, Cell Signaling Technology, 1:1000).

### 2.6 In vitro hydroxylation assay

In vitro hydroxylation assay was performed as previously described.^31^ Briefly, resin‐bound bacterially expressed oxygen‐dependent degradation domain of HIF1α (aa 401–603) or its P564A mutant were mixed with WT or AMPKα1^–/–^ cell lysate in NETN buffer. After mild agitation, the reaction mixtures were centrifuged and washed. The final resin‐bound proteins were dissolved in SDS buffer, followed by western blot with antibodies indicated.

### 2.7 In vitro kinase assay

The in vitro kinase assay was performed as described previously.^26^ Briefly, purified PHD2 protein was incubated for 30 min at 37°C in the absence or presence of recombinant AMPKα1β1γ1 /ATP/AMP in a kinase buffer. Reactions were stopped by adding SDS sample buffer, followed by western blot analysis of the samples.

### 2.8 Statistical analysis

Data are presented as mean ± SEM (standard error of the mean). The results were analysed with GraphPad Prism (GraphPad Software). Groups were considered significantly different at *p* values < .05. The Mann–Whitney test for non‐parametric variables and Student *t* test or one‐way analysis of variance (ANOVA) for parametric variables were used to identify statistically significant data. The statistical significance of correlations was determined by Pearson's correlation coefficient analysis.

## 3 RESULTS

### 3.1 AMPKα1^–/–^ mice display hypoferraemia and sequestration of iron

To investigate whether AMPKα1 deletion affected iron metabolism, we first measured serum iron. Levels of serum iron were reduced by 20% in AMPKα1^–/–^ mice relative to those of wild‐type (WT) mice (Figure S1A). In contrast, serum iron levels were similar in AMPKα2^–/–^ and WT mice. Furthermore, serum transferrin saturation (serum iron/total iron‐binding capacity [TIBC] ×100%) was lower in AMPKα1^–/–^ mice than in WT and AMPKα2^–/–^ mice (Figure S1B), reflecting the presence of iron deficiency in AMPKα1^–/–^ mice.

Consistent with these findings, serum ferritin, a highly and ubiquitous conserved protein playing a critical role in iron homeostasis by sequestering and storing iron in a non‐toxic, soluble form,^32^ was increased in AMPKα1^–/–^ mice, suggesting the presence of disturbed systemic iron balance (Figure S1C). Splenic macrophages phagocytise and degrade damaged and senescent erythrocytes to recycle iron, leading us to explore whether excess iron was deposited in the AMPKα1^–/–^ mice. Using Perls Prussian blue staining to detect iron deposition, we found more iron deposition in the spleen of AMPKα1^–/–^ mice compared with WT and AMPKα2^–/–^ mice (Figure S1D). Accordingly, quantification of iron concentrations showed that levels of iron in spleen in AMPKα1^–/–^ mice were approximately 3 times higher than those in WT and AMPKα2^–/–^ mice. In the liver, we also showed that visualised location of iron deposits within the liver of the AMPKα1^–/–^ mice (Figure S1E).

Because serum iron deficiency in AMPKα1^–/–^ mice may also be due to a defect in iron absorption from the duodenum into the circulation, we next examined iron retention in the duodenum. As shown in Figure S1F, Perls staining revealed prominent cellular iron deposition in duodenal enterocytes of AMPKα1^–/–^ mice, which need to be absorbed in the circulation. These findings suggest that AMPKα1 deficiency causes iron sequestration, thus contributing to dysfunction of iron homeostasis.

High hepcidin levels parallel low ferroportin levels in AMPKα1^–/–^ mice. Hepcidin is a master regulator of iron homeostasis, affecting the release of iron from the macrophages and duodenum into the blood.^10^, ^33^ We next investigated whether AMPKα1 deletion altered hepcidin expression. Because hepatocytes are the master producers of hepcidin, we examined hepcidin expression in the liver. The mRNA levels of hepcidin in the liver were upregulated in AMPKα1^–/–^ mice relative to levels in WT and AMPKα2^–/–^ mice (Figure S2A). ELISA analysis also confirmed increased hepcidin expression in serum from AMPKα1^–/–^ mice compared with that from WT and AMPKα2^–/–^ mice (Figure S2B).

As hepcidin accelerates internalisation and degradation of ferroportin, the only known cellular iron exporter,^34^ we next determined whether increased hepcidin levels lowered ferroportin levels in the spleen, liver and intestines. Western blot analysis showed decreased ferroportin levels in the spleen, liver and duodenum of AMPKα1^–/–^ mice (Figure S2C), suggesting that upregulation of hepcidin in AMPKα1^–/–^ mice leads to loss of ferroportin in spleen, liver and enterocytes.

### 3.2 Myeloid AMPKα1 contributes to neither abnormal iron sequestration nor reduced serum iron levels

In addition to hepatocytes, which are the major source of circulating hepcidin, other cell types like macrophages also express hepcidin mRNA. To exclude a potential contribution of myeloid cell to abnormal iron metabolism, AMPKα1^fl/fl^Lyz^+/–^ mice, which exhibited a myeloid‐specific deficiency of AMPKα1, were generated. Perls Prussian blue staining confirmed nearly no detectable iron deposition in the spleen, liver and duodenum of these mice (Figure S2D). There was also no difference of iron content in the spleen and liver between AMPKα1^fl/fl^Lyz^+/–^ and AMPKα1^fl/fl^Lyz^–/–^ mice (Figure S2D). Consistent with this finding, serum iron concentrations were not reduced in AMPKα1^fl/fl^Lyz^+/–^ mice compared with AMPKα1^fl/fl^Lyz^–/–^ mice (Figure S2E). Additionally, the content of hepcidin was also not changed (Figure S2F). Overall, these results indicate that the increased liver iron deposition and reduced serum iron levels observed in AMPKα1^–/–^ mice are likely not due to a dysfunctional myeloid system.

### 3.3 Liver‐specific AMPKα1 knockout mice display hypoferraemia and increased hepatic hepcidin expression

**FIGURE 1:**
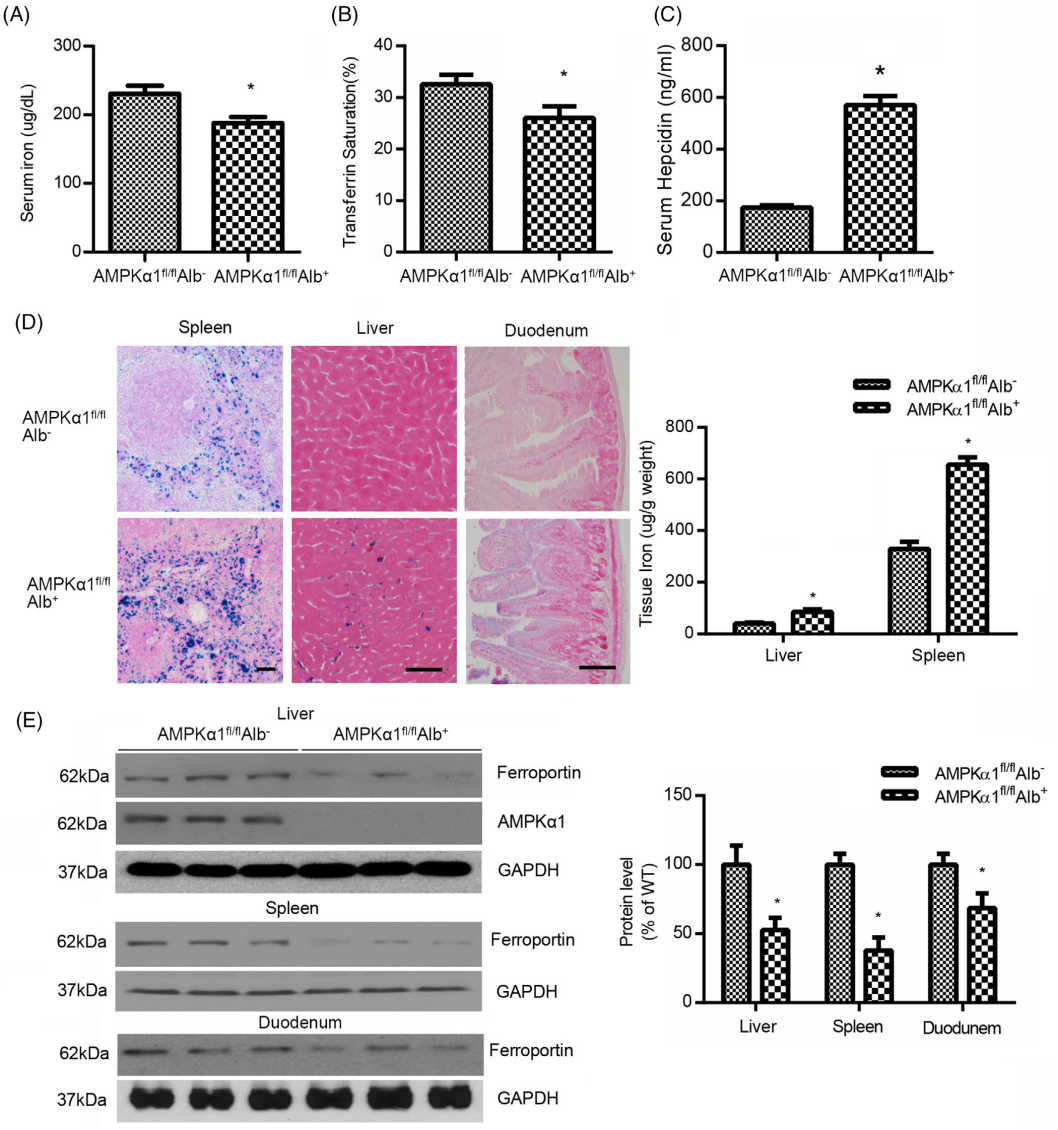
Liver‐specific AMPKα1 knockout mice display hypoferraemia and iron deposition. (A) Serum iron concentrations and (B) transferrin saturation in AMPKα1^fl/fl^Alb^+^ and AMPKα1^fl/fl^Alb^–^ mice. *p < .05 versus AMPKα1^fl/fl^Alb^–^ (*n* = 15). (C) Serum hepcidin level from AMPKα1^fl/fl^Alb^+^ and AMPKα1^fl/fl^Alb^–^ mice. *p < .05 versus AMPKα1^fl/fl^Alb^–^ (*n* = 10). (D) Perls Prussian blue staining results of spleen, liver and duodenum sections from AMPKα1^fl/fl^Alb^+^ and AMPKα1^fl/fl^Alb^–^ mice. Bar = 50 μm. (E) Western blot analysis of liver, spleen and duodenal ferroportin expression in AMPKα1^fl/fl^Alb^+^ and AMPKα1^fl/fl^Alb^–^ mice. GAPDH was used as the control. *p < .05 versus AMPKα1^fl/fl^Alb^–^ (*n* = 5)

Next, we generated liver‐specific AMPKα1 knockout (AMPKα1^fl/fl^Alb^+^) mice to explore the role of hepatic AMPKα1 in iron imbalance. Serum iron and transferrin saturation were reduced in AMPKα1^fl/fl^Alb^+^ mice relative to AMPKα1^fl/fl^Alb^–^ mice (Figure 1A, B), and hepcidin content in serum was upregulated in AMPKα1^fl/fl^Alb^+^ mice (Figure 1C). As shown in Figure 1D, Perls Prussian blue staining revealed significantly enhanced iron deposition in the spleen and liver of AMPKα1^fl/fl^Alb^+^ mice. To further confirm our results, AMPKα2^fl/fl^Alb^+^ mice, which were liver‐specific deficient in AMPKα2, were generated. There was no significant difference between AMPKα2^fl/fl^Alb^+^ and AMPKα2^fl/fl^Alb^–^ mice in serum iron levels and transferrin saturation. Perls Prussian blue staining also showed no statistic difference in iron deposition of the spleen and liver (Figure S3A, B), suggesting that liver‐specific AMPKα1 knockout mice, not AMPKα2 knockout mice, display iron sequestration and anaemia.

We further examined serum hepcidin levels and the expression of ferroportin in the spleen, liver and intestines from AMPKα1^fl/fl^Alb^–^ and AMPKα1^fl/fl^Alb^+^ mice. As shown in Figure 3E, decreased ferroportin levels were found in AMPKα1^fl/fl^Alb^+^ mice compared with AMPKα1^fl/fl^Alb^–^ mice. Consistently, no any differences in ferroportin expression were observed in the duodenum, liver and spleen from AMPKα2^fl/fl^Alb^+^ and AMPKα2^fl/fl^Alb^–^ mice (Figure S3C–E). These data further demonstrate that liver‐specific AMPKα1 deficiency can limit iron supply to erythropoiesis to develop anaemia of chronic disease via regulation of the hepcidin/ferroportin pathway.

### 3.4 AMPKα1 deficiency induces hepcidin expression by inhibiting HIF1α binding to the hepcidin promoter

To explore the molecular basis for AMPKα1‐deficiency–induced hepcidin expression, we first examined BMP/Smad signalling pathways. As shown in Figure S4A, there were no differences in levels of pSmad1 (Ser463/465)/Smad5 (Ser463/465)/Smad8 (Ser426/428) between WT and AMPKα1^–/–^ mice, indicating that Smad signalling does not contribute to the increased hepcidin expression in AMPKα1^–/–^ mice. Evidence indicates that STAT3 can regulate hepcidin expression by binding to the promoter of hepcidin antimicrobial peptide (HAMP), the gene encoding hepcidin.^16^ Using a luciferase assay in HepG2 cells, we found that AMPKα1 knockdown markedly increased the HAMP promoter activity. However, when the putative STAT3 binding site in the HAMP promoter was deleted, upregulation of the promoter activity by AMPKα1 knockdown was not blocked (Figure S4B); this suggests that the effects of AMPKα1 on hepcidin are not attributed to the STAT3 signalling pathway.

**FIGURE 2:**
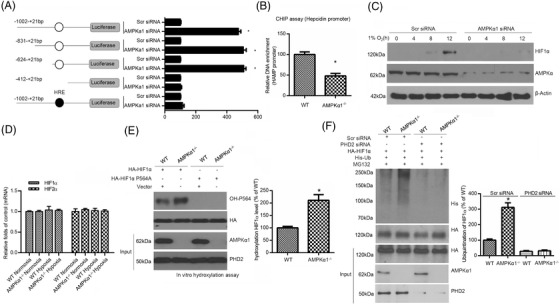
AMPKα1 deficiency upregulates hepcidin expression through hydroxylation of HIF1α. (A) HepG2 cells were pre‐transfected with Scr siRNA and AMPKα1 siRNA for 24 h, then transfected with various HAMP promoter truncation constructs or the deletion (HIF1α binding site) construct for 24 h. After this, luciferase activity was measured. *p < .05 versus Scr siRNA (*n* = 6). (B) ChIP assays using anti‐HIF1α antibody to amplify HAMP promoter in primary hepatocytes. *p < .05 versus WT (*n* = 5). (C) HepG2 cells were transfected with Scr siRNA or AMPKα1 siRNA. After 16 h, cells were exposed to hypoxia for different periods of time, as indicated, and then lysed and analysed by immunoblotting with HIF1α antibody. (D) AMPKα1^–/–^ and WT hepatocytes maintained in normoxia or hypoxia for 12h were subjected to real‐time PCR analysis for mRNA levels of HIF1α and HIF2α (*n* = 5). (E) In vitro HA‐HIF1α (amino acids 401–603) or P564A mutant proteins were separately added to cell lysates of AMPKα1^–/–^ and WT hepatocytes, followed by incubation for 90 min. The mixtures were then analysed for levels of HIF1α hydroxylation. *p < .05 versus WT (*n* = 5). (F) AMPKα1^–/–^ and WT hepatocytes transfected with Scr siRNA or PHD2 siRNA were transfected with different combinations of HA‐HIF1α and His‐Ub. At 16 h post‐transfection, cells were treated with 10 μM MG‐132 (proteasome inhibitor) for another 6 h and then subjected to immunoprecipitation with antibody against HA for HIF1α. The IP product was analysed by immunoblotting with His antibody to quantify ubiquitination of HIF1α. *p < .05 versus WT (*n* = 5)

To determine the mechanism whereby AMPKα1 deficiency enhanced HAMP promoter activity, we constructed a series of luciferase reporter plasmids with different lengths of HAMP promoter, including pGL3 (control), pGL3‐HAMP‐400 (–412 to +21 bp), pGL3‐HAMP‐600 (–624 to +21 bp), pGL3‐HAMP‐800 (–831 to +21 bp) and pGL3‐HAMP‐1000 (–1002 to +21 bp), and performed luciferase reporter assays. AMPKα1 knockdown markedly promoted the luciferase activity of 3 plasmids (pGL3‐HAMP‐600, pGL3‐HAMP‐800 and pGL3‐HAMP‐1000), but not the activity of pGL3 (control) or pGL3‐HAMP‐400, indicating that the region located between –624 and –412 bp is critical for hepcidin regulation under AMPKα1 deficiency (Figure 2A). We screened one candidate consensus hypoxia‐response element that was reported by Dr. Randall S. Johnson located at this specific fragment (–624 to –412 bp).^20^ To further validate our hypothesis, we deleted the putative binding site around –582 bp (5ʹ‐CAATG‐3ʹ) in pGL‐HAMP‐1000 to generate pGL‐HAMP‐1000‐Δ. The luciferase activity of this plasmid was abrogated regardless of co‐transfection with AMPKα1 small interfering RNA (siRNA) (Figure 2B). We further tested whether AMPKα1 deficiency disrupted HIF1α binding to this specific region in the HAMP promoter. Chromatin immunoprecipitation (ChIP) assays showed that HIF1α directly bound to this region in the HAMP promoter. However, the binding affinity of HIF1α for the HAMP promoter was dramatically decreased in AMPKα1‐deficient primary hepatocytes (Figure 2B). Collectively, these results indicate that HIF1α plays an essential role in AMPKα1 deficiency‐related hepcidin transactivation.

### 3.5 AMPKα1 deficiency suppresses HIF1α protein levels via promoting hydroxylation and ubiquitination of HIF1α in a PHD2‐dependent manner

The reduced DNA‐binding ability of HIF1α prompted us to explore whether AMPKα1 could directly regulate HIF1α expression. We knocked down AMPKα1 in HepG2 cells and detected a major decrease in HIF1α at the protein level (Figure 2C). Of note, there were no changes in HIF1α and HIF2α mRNA levels between WT and AMPKα1^–/–^ primary hepatocytes under hypoxic or normoxic conditions (Figure 2D), indicating that AMPKα1 affects the protein levels of HIF1α in a post‐transcriptional manner. To explore the mechanism how AMPKα1 deficiency downregulated HIF1α protein levels at the post‐transcriptional level, we explored the possibility that AMPKα1 deficiency promoted hydroxylation and ubiquitination of HIF1α, two modifications critical for HIF1α proteasomal degradation. Using in vitro hydroxylation assays, we found that HIF1α hydroxylation at Pro564 was significantly increased when cell lysates from AMPKα1^–/–^ cells was added (Figure 2E). As a control, AMPKα1 did not alter hydroxylation of mutated HIF1α lacking this hydroxylated proline residue (HIF1α P564A).

Ubiquitinated HIF1α was increased in AMPKα1^–/–^ primary hepatocytes treated with the proteasome inhibitor MG‐132, and depletion of PHD2 impaired HIF1α ubiquitination that was enhanced by AMPKα1 deficiency (Figure 2F). Overall, these results suggest that AMPKα1 deletion downregulates HIFα protein levels via accelerating hydroxylation and ubiquitination of HIF1α, which possibly depends on the PHD2 pathway.

### 3.6 AMPKα1 forms a complex with PHD2

**FIGURE 3:**
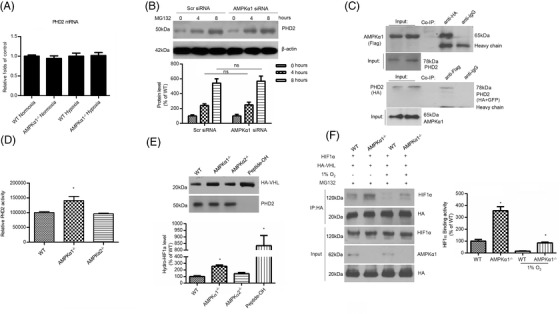
AMPKα1 interacts with PHD2 and inhibits PHD2 activity. (A) AMPKα1^–/–^ and WT hepatocytes maintained in normoxia or hypoxia were subjected to real‐time PCR analysis to determine mRNA levels of PHD2. (B) HepG2 cells were transfected with Scr siRNA or AMPKα1 siRNA. At 16 h post‐transfection, cells were treated with the proteasome inhibitor MG‐132 for the indicated times. Western blot analysis was performed to evaluate PHD2 protein expression. (C) Flag‐AMPKα1 and HA‐PHD2 in whole cell lysates were Co‐IP by the appropriate primary antibodies and subjected to western blot analysis to detect binding of AMPKα1 and PHD2. (D) AMPKα1^–/–^ or WT hepatocytes were lysed and assayed for PHD protein activity. *p < .05 versus WT (*n* = 11). (E) AMPKα1^–/–^, AMPKα2^–/–^ or WT hepatocyte lysates were added to immobilise HIF1α peptide containing proline 564. Peptides were washed, and HA‐pVHL was allowed to bind to the HIF1α peptide. Bound HA‐pVHL was measured by western blotting using anti‐HA. Hydroxylated synthetic HIF1α peptide (Peptide‐OH) was the positive control. *p < .05 versus WT (*n* = 5). (F) AMPKα1^–/–^ or WT hepatocytes were transfected with different combinations of HIF1α and HA‐VHL. At 24 h post‐transfection, cells were treated with 10 μM MG‐132 and maintained in normoxia or exposed to hypoxia for another 8 h, after which they were lysed. The protein extracts were immunoprecipitated with antibody against HA to quantify VHL. *p < .05 versus WT (*n* = 5)

We next investigated whether the effect of AMPKα1 deficiency on HIFα hydroxylation and ubiquitination occurred directly through increasing PHD2 level. Using a qPCR assay, we found no differences in PHD2 mRNA levels in WT and AMPKα1^–/–^ primary hepatocytes under normoxic or hypoxic conditions (Figure 3A). We also determined the half‐life of endogenous PHD2 protein in AMPKα1‐deficient HepG2 cells. As shown in Figure 3B, AMPKα1 deficiency had no effects on PHD2 protein stability. These data indicate that AMPKα1 does not influence PHD2 expression.

We then asked whether AMPKα1 could form a complex with PHD2 and disrupt PHD2‐mediated HIFα hydroxylation. Immunoprecipitation of AMPKα1 followed by probing for PHD2 or vice versa demonstrated that AMPKα1 physically associated with PHD2 (Figure 3C), indicating their coexistence in the same complex.

### 3.7 AMPKα1 inhibits PHD2‐dependent hydroxylation activity

Next, we used a quantitative PHD2 activity assay to determine whether the binding of AMPKα1 with PHD2 could alter PHD2 hydroxylation activity.^35^ Peptides corresponding to residues 556–574 of HIF1α were immobilised and then incubated with protein lysates. The amount of Pro564 hydroxylation was measured by the specific HIF1α hydroxypro564 antibody. Interestingly, a significant increase in PHD2 activity was observed under AMPKα1 deletion in primary hepatocytes (Figure 3D) compared with WT or AMPKα2^–/–^ cells.

It is well known that hydroxylated HIF1α exhibits highly affinity for VHL protein, a component of the ubiquitin E3 ligase complex, which causes polyubiquitination of HIF1α for proteasomal degradation. We used primary hepatocyte lysates containing PHD2 to hydroxylate a synthetic peptide of HIF1α (residues 556–574), then assayed PHD2 activity using the pVHL capture method. As shown in Figure 3E, AMPKα1^–/–^ hepatocytes exhibited higher levels of captured haemagglutinin (HA)‐VHL protein than WT or AMPKα2^–/–^ hepatocytes. Furthermore, a co‐immunoprecipitation experiment showed that AMPKα1 deficiency significantly promoted the interaction between VHL and HIF1α under both hypoxic and normoxic conditions (Figure 3F). Taken together, these data indicate that AMPKα1 deficiency enhances PHD2 activity, which in turn recruits more hydroxylated HIFα to VHL for degradation.

### 3.8 AMPKα1 phosphorylates PHD2 at ser61 and ser136

**FIGURE 4:**
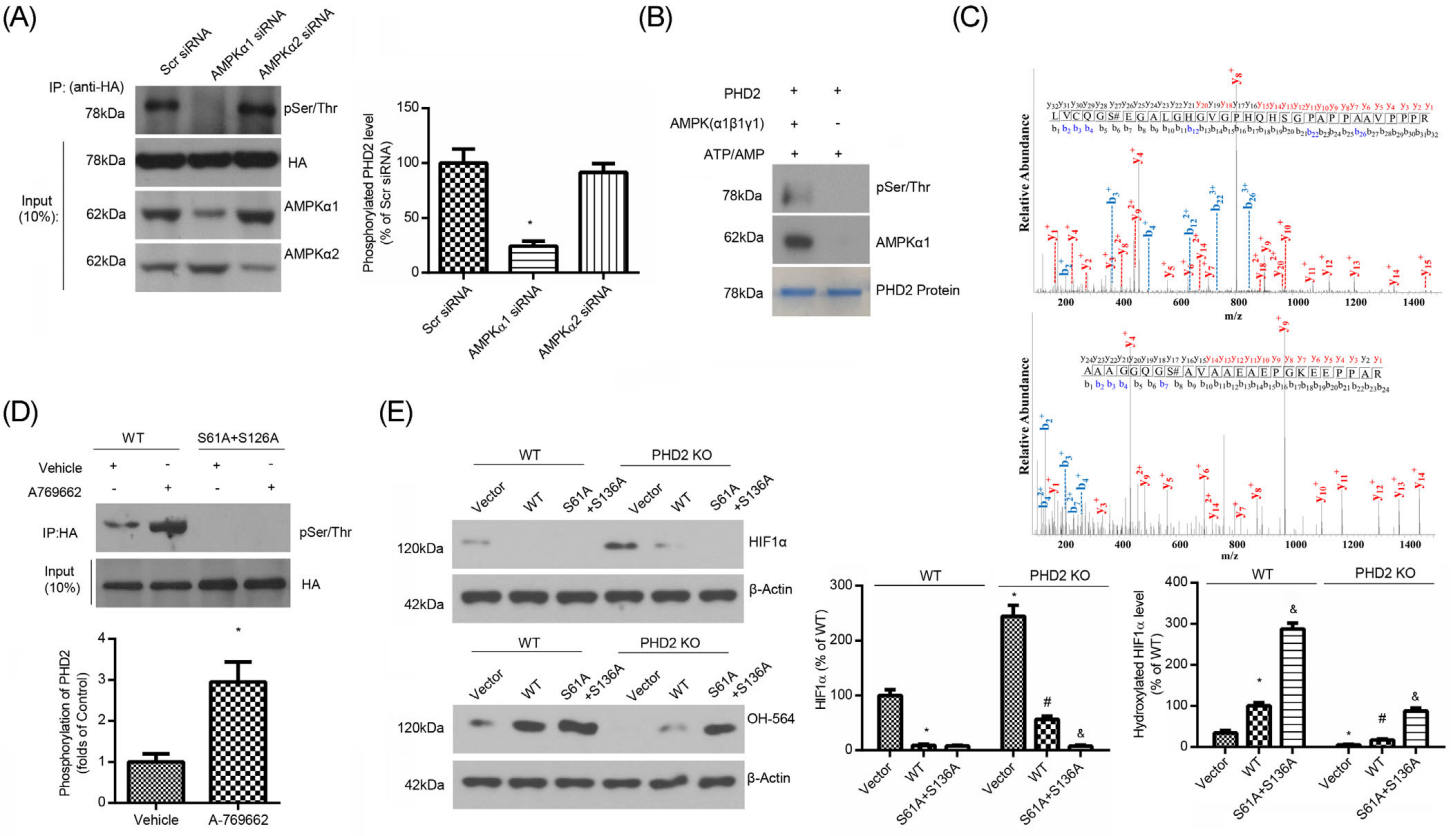
AMPKα1 phosphorylates PHD2 at Ser61 and Ser136. (A) HepG2 cells, pre‐transfected with Scr siRNA, AMPKα1 siRNA, or AMPKα2 siRNA, were then transfected with HA‐PHD2. At 24 h post‐transfection, cells were subjected to IP with antibody against HA to determine the phosphorylation of PHD2. *p < .05 versus WT (*n* = 5). (B) In vitro kinase assay with and without AMPKα1β1γ1 complex kinase. (C) The ETD MS/MS spectrum of trypsin peptide 56–87 (LVCQGSEGALGHGVGPHQHSGPAPPAAVPPPR) and 129–152 (AAAGGQGSAVAAEAEPGKEEPPAR) from phosphorylated PHD2 showing phosphorylation at Ser61 and Ser136. (D) HepG2 cells were transfected with HA‐tagged WT and site‐directed mutants of PHD2 and then treated with or without A‐769662 for 6 h. Serine phosphorylation of PHD2 was determined by IP assay. *p < .05 versus WT (*n* = 5). (E) HA‐tagged WT or double‐site–directed mutants of PHD2 were transfected into WT or PHD2 knockout HepG2 cells, and then cells were treated with 10 μM MG‐132 for 8 h. The levels of hydroxylated HIF1α were determined by western blot assay. *p < .05 versus Vector (WT); ^#^ *p* < .05 versus WT (WT) (*n* = 5), ^&^ *p* < .05 versus WT (PHD2 KO) (*n* = 5)

Because AMPK is a threonine/serine protein kinase, we speculated that AMPKα1 controls PHD2 hydroxylation activity by phosphorylating PHD2. To test this hypothesis, we first measured total serine phosphorylation of PHD2 in HepG2 cells after transfection with AMPKα1 or AMPKα2 siRNA, respectively. As shown in Figure 4A, knockdown of AMPKα1, but not AMPKα2, abolished serine phosphorylation of PHD2.

To further determine whether AMPKα1 could directly phosphorylate PHD2, we conducted an in vitro AMPK kinase assay. As depicted in Figure 4B and S5, AMPKα1β1γ1, not AMPKα2β1γ1, significantly increased phosphorylation of PHD2, suggesting that AMPKα1 functions as the upstream kinase and directly phosphorylates PHD2.

Next, we determined the serine residue(s) of PHD2 that can be phosphorylated by AMPKα1. Liquid chromatography (LC)–mass spectrometry (MS)/MS analysis was conducted in parallel on trypsin digests of purified native PHD2 after in vitro phosphorylation by AMPKα1. It was unambiguously identified Ser61 and Ser136 in digests of the PHD2 phosphorylated sample (Figure 4C). In HepG2 cells transfected with WT PHD2 plasmid, treatment with A‐769662, a potent activator of AMPK, led to PHD2 serine phosphorylation. We thus generated a double‐site–directed mutant construct, the PHD2 S61A + S136A plasmid. As expected, A‐769662 treatment did not further increase PHD2 phosphorylation in cells transfected with the PHD2 S61A + S136A double mutant (Figure 4D). Together, these data indicate that Ser61 and Ser136 are required for phosphorylation of PHD2.

Next, we wondered whether the phosphorylation of PHD2 at Ser61 and Ser136 by AMPKα1 could affect the hydroxylation activity of PHD2. Indeed, overexpression of mutated PHD2 (S61A + S136A) in either WT or PHD2‐deleted HepG2 cells markedly upregulated the Pro564 hydroxylation level of HIF1α, together with decreased HIF1α levels, compared with forced expression of PHD2 (Figure 4E). Collectively, these data indicate that phosphorylation of PHD2 at Ser61 and Ser136 disrupts PHD2‐dependent hydroxylation activity.

### 3.9 PHD2 inhibition abolishes hypoferraemia and iron sequestration in AMPKα1‐deficient mice

**FIGURE 5:**
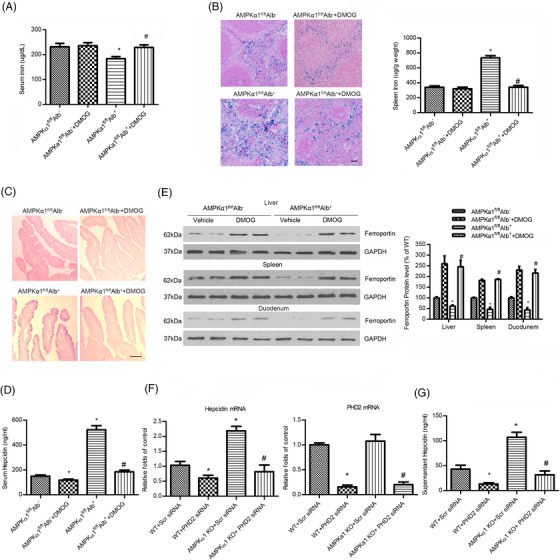
PHD2 inhibition abolishes hypoferraemia and iron sequestration in AMPKα1^fl/fl^Alb^+^ mice. AMPKα1^fl/fl^Alb^+^ and AMPKα1^fl/f^Alb^–^ mice were received intraperitoneal injections of DMOG at 8 mg/mouse each day for 4 weeks (*n* = 8). (A) Serum iron measurements and (B) Perls Prussian blue staining of spleen sections. *p < .05 versus AMPKα1^fl/fl^Alb^–^ mice; ^#^ *p* < .05 versus AMPKα1^fl/fl^Alb^+^ mice (*n* = 8). (C) Perls Prussian blue staining of duodunum sections. Bar = 50 μm. (D) Serum hepcidin level from these mice. *p < .05 versus AMPKα1^fl/fl^Alb^–^ mice; ^#^ *p* < .05 versus AMPKα1^fl/fl^Alb^+^ mice (*n* = 8). (E) Western blot analysis of ferroportin expression in the liver, spleen and duodenum. (F) Real‐time PCR analysis of hepcidin and PHD2 mRNA of AMPKα1^–/–^ and WT hepatocytes transfected with Scr siRNA or PHD2 siRNA. (G) ELISA analysis of hepcidin protein expression of AMPKα1^–/–^ and WT hepatocytes transfected with Scr siRNA or PHD2 siRNA. *p < .05 versus AMPKα1^–/–^ hepatocytes; ^#^ *p* < .05 versus AMPKα1^–/–^ hepatocytes plus Scr siRNA (*n* = 5)

Because AMPKα1 directly phosphorylated PHD2 at Ser61 and Ser136 and suppressed PHD2‐dependent hydroxylation on HIF1α and subsequent regulation of hepcidin, we next tested whether inhibition of PHD2 hydroxylation could ameliorate abnormal iron metabolism in liver‐specific AMPKα1‐deficient mice. Adult AMPKα1^fl/fl^Alb^+^ mice were treated for 4 weeks with dimethyloxalylglycine (DMOG), a 2‐oxoglutarate analogue that inhibits PHD2 hydroxylase activity. As depicted in Figure 5A–C, DMOG significantly rescued serum iron levels and reduced iron deposition in liver‐specific AMPKα1‐deficient mice. DMOG also significantly reduced the elevated serum hepcidin levels, and normalised intestinal, liver and spleen ferroportin expression in AMPKα1^fl/fl^Alb^+^ mice (Figure 5D, E). Furthermore, PHD2 silencing achieved by siRNA transfection markedly reduced the upregulated hepcidin mRNA and supernatant levels in AMPKα1^–/–^ hepatocytes in vitro (Figure 5F, G). Overall, these results indicate that iron dyshomeostasis in AMPKα1^–/–^ mice is attributed to increased PHD2‐related hydroxylation activity.

### 3.10 Clinical relevance of AMPKα1/PHD2/HIF1α/hepcidin axis

**FIGURE 6:**
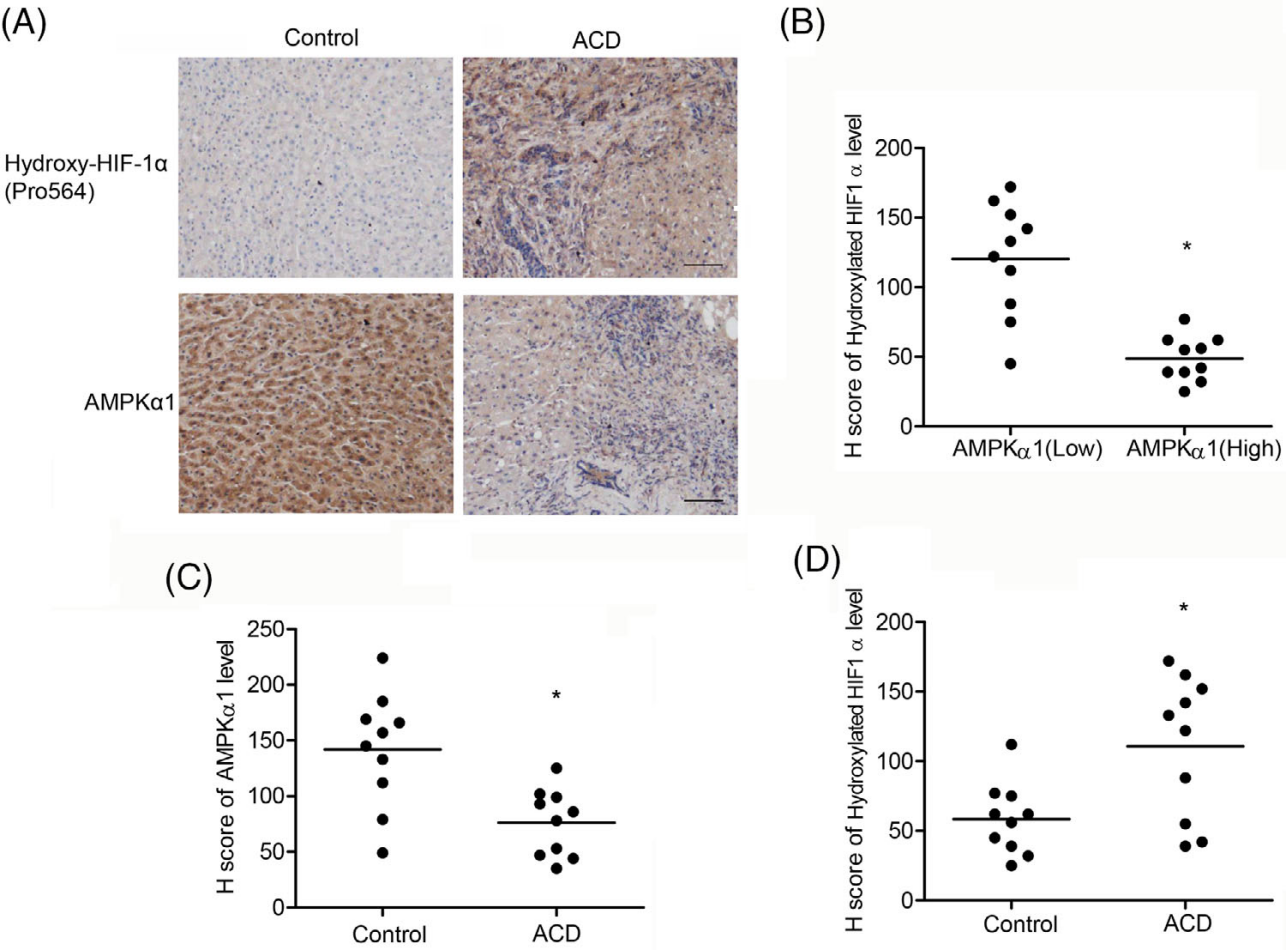
Clinical relevance of AMPKα1/PHD2/HIFα/hepcidin/ferroportin axis. (A) Immunostaining of AMPKα1 and hydroxyl‐HIF1α (Pro564) in sections of the liver from patients with ACD (*n* = 10) and normal control (*n* = 10). Bar = 50 μm. (B) The AMPKα1 levels were negatively correlated with hydroxyl‐HIF1α (Pro564) expression in the human liver specimens. (C and D) AMPKα1 and hydroxyl‐HIF1α (Pro564) expression levels were quantified by IHC from patients with ACD (*n* = 10) and normal control individuals (*n* = 10)

Finally, we examined whether the AMPKα1/PHD2/HIFα/hepcidin/ferroportin axis in vivo identified was clinically relevant to iron dyshomeostasis in anaemia of advanced malignant tumour. Liver tissues from adult patients with anaemia of chronic disease displayed a clear downregulation of AMPKα1 in parallel with increased levels of hydroxy‐HIF1α (Figure 6A, B). Statistically, the AMPKα1 levels were downregulated in the liver samples from anaemia of chronic disease (Figure 6C). Consistently, elevated expression of hydroxy‐HIF1α in the human liver biopsy was correlated with anaemia of chronic disease (Figure 6D). Altogether, our results suggest that AMPK targets on PHD2/HIF1α to modulate iron metabolism.

## 4 DISCUSSION

In this study, we found that deletion of AMPKα1, but not AMPKα2, caused abnormal iron metabolism, and more importantly, hepatic AMPKα1 deficiency, but not myeloid AMPKα1 deficiency, caused decreased serum iron levels and prominent iron deposition in macrophages, in parallel with higher level of hepcidin and lower expression of ferroportin. We found that AMPKα1 directly bound to and phosphorylated PHD2 at Ser61 and Ser136, thus inhibiting PHD2 hydroxylation activity on HIF1α. Taken together, our results indicate that AMPKα1 may play an essential role in maintaining systemic iron homeostasis by regulating PHD2/HIFα/hepcidin/ferroportin signalling.

AMPK participates in the regulation of eryptosis during energy depletion. Marc Foretz et al. reported that AMPKγ1^–/–^ mice could cause iron accumulation because of compensatory splenic erythropoiesis and erythrophagocytosis.^36^ Interestingly, previous work showed that AMPKα1^–/–^ mice also manifested splenomegaly and anaemia via defective autophagy‐dependent mitochondrial clearance.^37^ Erythrocyte instability in AMPKα1^–/–^ mice surely may alter system iron homeostasis^38^; however, in this study, we discovered that AMPKα1^fl/fl^Alb^+^ mice (liver‐specific AMPKα1 deficiency), which could not cause a defect in erythrocytes (Figure S6), had a similar phenotype as global AMPKα1^–/–^ mice, such as lower serum iron levels, in association with iron sequestration in the spleen and liver. These results explained why hepatic AMPKα1 played a decisive role in systemic iron regulation but also displayed less non‐haeme iron deposition in the spleen and liver than that in global AMPKα1^–/–^ mice.

The liver‐derived hepcidin is central to systemic iron homeostasis regulation. It regulates and is in turn regulated by systemic iron levels.^15^ Our data showed that hepatic AMPKα1 deletion led to increased hepcidin expression and serum iron deficiency. Hepcidin is well known to be regulated at the transcriptional level by BMPs, inflammatory cytokines, iron and hypoxia. A previous study reported that metformin suppressed BMP6‐induced hepcidin.^39^ Our results, however, revealed that AMPKα1 deficiency did not change the protein levels of p‐Smad1/Smad5/Smad8, the critical regulator of the BMP6 pathway. It has been reported that AICAR and metformin are not direct AMPK activator, phosphorylation of Thr172 by metformin may indicate involvement of AMP stress‐induced unspecific AMPK activation, possibly activating other AMP‐dependent kinases,^40^ which cause activation of BMP6 signalling pathway. Furthermore, when the putative STAT3 binding sites in the HAMP promoter plasmid were deleted, the suppression of AMPKα1 on the hepcidin promoter was not altered. These results strongly suggest that AMPKα1‐deficiency–induced hepcidin expression occurs through neither the BMP/SMAD pathway nor the STAT3 inflammatory pathway.

Liver hypoxia is a strong repressor of hepcidin expression.^17^ Hypoxia can override the upregulation of hepcidin during chronic liver inflammation. Recent studies indicate that in the absence of hypoxia, HIF1α activation in hepatocytes could suppress hepcidin expression and promote ferroportin expression in intestine and macrophages.^17^, ^41^ Interestingly, using a truncation luciferase reporter assay, we found that AMPKα1 deficiency upregulated the transcriptional activity of hepcidin via inhibiting HIF1α binding to the HAMP promoter. Our data are consistent with results of Peyssonnaux et al.,^20^ although Volke and colleagues and Braliou et al. reported that HIF‐1 may not affect hepcidin expression via a direct transcriptional suppression.^42^, ^43^ The discrepancy between different groups might be specifically due to the different source of cells, and primary liver cells are close to normal physical condition. Consistent with these findings above, the expression of HIF1α and HIF2α in the liver of AMPKα1^–/–^ mice was significantly decreased compared with that in WT and AMPKα2^–/–^ mice. While in the mouse embryonic fibroblasts (MEFs), previous researches showed that elevated HIF1α protein levels following AMPKα1/α2 deletion independent of mRNA levels.^44^, ^45^ It is clear that AMPK α1 and α2 subunits have distinct biological functions; they may cooperate or antagonise each other to maintain the homeostasis of cell signalling. Loss of interplay between α1 and α2 isoforms and differences in cell species may mediate this opposite effect. More importantly, our data showed that hepatic AMPKα1 altered HIF1α protein levels through PHD2‐dependent hydroxylation. In addition to HIF1α, even HIF2α does not regulate hepcidin directly in the liver, but rather by affecting erythropoietic activity to downregulate hepcidin expression.^46^ All of these data suggest that AMPKα1 may play an essential role in HIF1α‐related HAMP transcriptional inhibition.

Hepcidin is a critical regulator of systemic iron homeostasis and mediator of anaemia of inflammation. Chronic inflammatory diseases, including cancer, chronic kidney disease and rheumatologic diseases are associated with abnormally high plasma concentrations of hepcidin.^47^ In anaemia of chronic disease, hepcidin production is increased, and this may account for the defining feature of this condition as sequestration of iron in macrophages. Previous research reported that hepcidin excess developed anaemia and low level of serum iron by shunting iron away from erythropoiesis and sequestering it in the liver.^48^ All these phenotypes are consistent with our phenotype that was caused by high hepcidin level. More importantly, we demonstrated that in mice with global or live‐specific AMPKα1 deficiency, ferroportin loss controlled by increased hepcidin decreased iron transfer to plasma from macrophages and from absorptive enterocytes that recycle the iron of senescent erythrocytes, which then caused anaemia of chronic disease. Interestingly, Roy et al. found that hepcidin‐transgenic mice displayed hypoferraemia and iron accumulated in tissue macrophages, whereas a relative paucity of iron was found in the liver.^49^ These hepcidin‐transgenic mice showed a dramatic increase in the level of hepcidin without inflammation. Consistently, young mice with deficiency of TMPRSS6 (coding for matriptase‐2, a liver transmembrane serine protease) causes a hair loss and an iron deficiency anaemia phenotype, characterised by upregulation of hepcidin and blocking iron export into plasma from intestinal.^50^, ^51^ However, adult hepatic AMPKα1‐deficient mice displayed mild systemic inflammation (data not shown). This chronic inflammation increases serum hepcidin content and results in defective iron reuse from body stores, which is highly consistent with the human pathological condition.^10^ In addition, the storage of iron in spleen and liver is significantly decreased in Roy's model, which is not consistent with the pathophysiological process of anaemia of inflammation. Tissue macrophages are capable of processing and releasing iron and are implicated in every step of iron recycling and metabolism.^52^ High level of hepcidin production may lead to sequestration of iron in tissue macrophages. The increased deposition of iron in liver macrophage is attributed to sealing off the aisle that releases iron for erythropoiesis by high hepcidin content in the AMPKα1 knockout condition. Further studies will be of interest to investigate the double deletion hepcidin in AMPKα1^–/–^ mice to confirm systemic iron regulation in anaemia of chronic disease.

Inhibition of PHDs represses expression of hepcidin in hepatoma‐derived cells.^43^ Reduced PHD2 activity has also been demonstrated to be involved in maintaining cellular iron homeostasis and neuronal viability.^53^ In our study, AMPKα1‐depleted hepatocytes exhibited significantly increased PHD2 activity. More interestingly, inhibition of PHD2 can prevent AMPKα1‐deficiency–induced abnormal hepcidin/ferroportin expression and iron metabolism. PHD2 has been recently recognised as the major regulator of multiple physiological and pathological responses, including pulmonary hypertension, stroke, myocardial ischemic injury and cancer.^54^, ^55^, ^56^, ^57^ It remains to be determined whether the AMPKα1/PHD2/HIF1α pathway is also involved in the progression of these diseases. Further studies should investigate whether AMPKα1‐related PHD2 modification affects the pathogenesis of other diseases besides iron metabolic homeostasis. Additionally, the effects of PHD2 inhibitor in vivo could be also explained by alternative mechanisms: PHD2 inhibitor increases erythropoietin (EPO) production, causing an increase in erythroferrone (ERFE) and the following hepcidin suppression.^58^ In duodenum, PHD2 inhibitor would cause a direct stabilisation of HIF2a and an increase in ferroportin mRNA level.^59^ This is a limitation for interpreting the in vivo results by PHD2 inhibitors. Further research needs to use liver‐specific AMPKα1 and PHD2 double deficiency mice to explore and address this possibility.

Iron in the form of haeme and iron–sulphur clusters are cofactors for PHD2 involved in a host of regulatory functions.^35^ In AMPKα1‐deficient mice, AMPKα1 deficiency accelerated PHD2‐dependent hydroxylation of HIF1α and subsequent regulation of hepatic hepcidin‐related iron signalling. This iron may be delivered to the Fe(II)‐dependent prolyl and asparaginyl hydroxylases regulating hydroxylation of HIF1α. It may form a feedforward pathway in PHD2‐related iron regulation. It has also been reported that high dietary iron could activate AMPK activity in liver through redox signalling and decrease LKB1 acetylation.^60^ Here, we provide evidence that, in AMPKα1‐deficient mice, AMPKα1 inactivation caused lower serum iron levels and more iron sequestration in the spleen and liver. All this evidence suggests that activated AMPK may provide an early signal to protect mice from iron‐mediated liver injury after high iron insult.

In summary, the present study has demonstrated for the first time, to our knowledge, that AMPKα1 regulates iron metabolism via the inhibitory effects on the PHD2/hepcidin axis. These findings, therefore, identify AMPKα1 as a potentially important therapeutic target for treating anaemia of chronic disease.

## CONFLICT OF INTEREST

The authors declare no conflict of interest.

## Supporting information

Supporting Information
