# Corrigendum notice

**DOI:** 10.1002/ctm2.1306

**Published:** 2023-06-14

Wang C, Zhang W, Xu W, et al. AMP‐activated protein kinase α1 phosphorylates PHD2 to maintain systemic iron homeostasis. Clin Transl Med. 2022 May;12(5):e854. https://doi.org/10.1002/ctm2.854.

In the originally published article by Cheng Wang et al. (2022), Ming‐hui Zou, Georgia State University, was not properly included as the corresponding author. Related grants from NIH (HL089920, HL137371, and HL142287) which were not properly acknowledged in the initial version of this article. The corrected authorship and acknowledgements are as the followings:

Cheng Wang^1,2,3,4,*^, Wencheng Zhang^5^, Wenjing Xu^1^, Zhaoyu Liu^6^, Kai Huang^1,2,*^, Ming‐Hui Zou^4,*^

^1^Clinic Center of Human Gene Research, Union Hospital, Tongji Medical College, Huazhong University of Science and Technology, Wuhan, China

^2^Hubei Key Laboratory of Metabolic Abnormalities and Vascular Aging, Tongji Medical College, Huazhong University of Science and Technology, Wuhan, China

^3^Department of Rheumatology, Union Hospital, Tongji Medical College, Huazhong University of Science and Technology, Wuhan, China

^4^Center for Molecular and Translational Medicine, Georgia State University, Atlanta, Georgia, USA

^5^Department of Cardiology, Qilu Hospital, Cheeloo College of Medicine, Shandong University, Jinan, China

^6^Department of Cardiology, Sun Yat‐sen Memorial Hospital, Sun Yat‐sen University, Guangzhou, China

The author apologizes for this error.

