# Supplementary material for: AMP‐activated protein kinase α1 phosphorylates PHD2 to maintain systemic iron homeostasis
Source: Clin Transl Med. 2022 May 11;12(5):e854. doi: 10.1002/ctm2.854 (PMC9091988; doi:10.1002/ctm2.854)
Supplement: Supplementary file 1 — Supporting Information [file CTM2-12-e854-s001.docx]

**Supplementary Methods**

**LC-MS/MS analysis**

Protein disulfides were reduced by 5 mM DTT at 37 °C for 60 minutes and alkylated with 14 mM iodoacetamide at RT for 25 min in the dark. Iodoacetamide was quenched by 10 mM DTT at RT for 30 min. The sample was diluted 2-fold with 100 mM ammonium bicarbonate (NH_4_HCO_3_) and then digested with trypsin (Promega) at 37 °C overnight. Formic acid (FA) was added to adjust the pH of the sample to be less than 2, and the peptides were purified via stage tip.

Purified and dried peptides were dissolved in loading buffer with 5% ACN and 4% FA and analyzed by a hybrid dual-cell quadrupole linear ion trap-Orbitrap mass spectrometer (LTQ Orbitrap Elite, Thermo Fisher Scientific, with Xcalibur 3.0.63 software) equipped with a Dionex UltiMate 3000 HPLC system (Thermo Fisher Scientific). For each cycle, one full MS scan (resolution: 60,000) in the Orbitrap at 106 AGC target was followed by peptide fragmentations with the high-energy collision dissociation (HCD) at 38% normalized collision energy for the 15 most intense ions. The selected ions were excluded from further analysis for 90 seconds, and ions with one or unassigned charge were excluded for fragmentation.

Peptide identification was performed by SEQUEST (version 28) ^1^against a database consisting of sequences from all human proteins (*Homo sapiens*) downloaded from the UniProt. The following parameters were used for the database search: 20 ppm precursor mass tolerance and 0.025 Da product ion mass tolerance; fully digested with trypsin; up to 3 missed cleavages; variable modifications: oxidation of methionine (+15.9949), phosphorylation of serine, threonine, and tyrosine (+79.9663); fixed modifications: carbamidomethylation of cysteine (+57.0214). The target-decoy method^2^, in which each sequence from a protein was listed in both forward and reverse orders, was employed to evaluate the false discovery rates (FDRs) of peptide identifications, and peptides were considered identified with an FDR less than 1%. ModScore was used to evaluate the possibility of the phosphorylation site localization, and sites with a Modscore >19 (*p* < 0.01) were considered to be well localized^3^. The phosphorylated peptides were further manually checked.

**ELISA for determination of ferritin levels**

A mouse ferritin ELISA kit (Abcam, Cambridge, UK) was used for quantification of ferritin levels in mice. The absorbance of the final reaction mixture was measured at 450 nm. A calibration curve was used as instructed in the kit to determine the ferritin levels in these samples.

**Serum hepcidin quantification**

Serum hepcidin were detected by using the hepcidin murine complete ELISA kit (Intrinsic Lifesciences, La Jolla, CA, US), according to the manufacturer’s instruction.

**Real-time quantitative polymerase chain reaction (RT-qPCR)**

Total RNA from liver was isolated using TRIzol (Invitrogen), followed by synthesis of double-stranded cDNA from the total RNA using the iScript cDNA Synthesis Kit (Bio-Rad, Hercules, CA, US). Real-time PCR analysis was performed using the SYBR Green Supermix (Bio-Rad). Amplification conditions were as follows: 95 °C for 10 min, followed by 40 cycles of 95 °C for 15 s and 60 °C for 30 s. The mRNA quantity of each transcript was calculated relative to the expression of the housekeeping gene GAPDH.

**Immunoprecipitation**

Briefly, 300 µg of protein extracts were incubated with the indicated antibodies or unspecific IgG at 4 °C overnight, and protein-A/G agarose was added for another 2–3 hours at 4 °C. The immunoprecipitates were pelleted by centrifugation at 5,000 rpm for 4 min and washed 3 times with RIPA lysis buffer. The pellets were suspended in sodium dodecyl sulfate (SDS) gel loading buffer and subjected to western blot assays.

**ChIP assay**

ChIP assay was treated according to the procedure^4^. Cells were cross-linked with 1% formaldehyde, then harvested and sonicated to generate DNA fragments of 0.2–1 kb. Lysates were centrifuged. Supernatants were immunoprecipitated with indicated antibodies or an IgG. Finally, DNA was purified using the QIAquick PCR purification kit (Qiagen). Purified DNA was analyzed by real-time PCR with specific primers for HAMP promoter.

**Erythrocyte reinfusion and clearance test**

*In vivo* RBC tracking was performed as reported previously with modifications^5^. Briefly, RBCs of donor mice were biotin-labeled *in vivo* by tail vein injection of N-succinimidyl-6-[biotinamido] hexanoate, as described above. One hour after biotin infusion, blood was collected from donor mice and placed in tubes containing heparin as an anticoagulant. A small aliquot of labeled RBCs was incubated with streptavidin-phycoerythrin and analyzed by flow cytometry to ensure that at least 95% of the blood cells were labeled. Blood was washed and resuspended in sterile saline, and 100 μl of RBC suspension was infused into each recipient mouse by tail vein injection. Initial postinfusion blood samples were obtained after 30 min and analyzed by flow cytometry. The typical initial percentage of biotin-labeled RBCs (recorded as day 0%) was greater than 5% in recipient mice. Seven days after blood reinfusion, the remaining labeled red cells were determined (recorded as day 7%). The red cell clearance rate was calculated as follows: clearance rate ((day 7% − day 0%)/day 0%) 100%.

**Transient transfection and luciferase assay**

Full-length 1-kb fragments of the human HAMP (hepcidin antimicrobial peptide) promoter, as well as various truncations, were cloned into the pGL3-Basic Vector (Promega) containing the firefly luciferase reporter (luc) gene. A putative HRE CACGTG motif was deleted by using the QuikChange Site-Directed kit (Stratagene, San Diego, CA, US) as indicated by the manufacturer. The deletion in the corresponding plasmid pGL3-HAMP was verified by sequencing. HepG2 cells were transfected with HAMP promoter luc plasmid or control plasmid containing the Renilla luciferase gene under control of the cytomegalovirus (CMV) promoter by using Lipofectamine 3000 transfection reagent (Thermo Fisher Scientific, Waltham, MA, US) according to the manufacturer’s instructions. Cells were then lysed in Passive Lysis Buffer (Promega), and cellular extracts were analyzed using the Dual-Luciferase-Reporter assay system (Promega) and a Centro LB 960 luminometer (Berthold Technologies, Bad Wildbad, Germany). For siRNA-mediated knockdown or plasmid transfection, HepG2 cells were transfected with siRNA or indicated plasmid for 24 h and then transfected with a HAMP promoter luc plasmid and a Renilla luciferase construct. After another 24 h, cell luciferase activity was measured.

**Plasmids**

Plasmid full-length cDNAs encoding human PHD2 and AMPKα1 were obtained by PCR using cDNA purified from HEK293T cells. Point mutations of PHD2 were performed by a PCR-based site-directed mutagenesis method using Pfu polymerase (Stratagene). DNA fragments amplified by PCR were verified by sequencing (Invitrogen, Shanghai, China). Expression plasmids for various proteins were constructed in the pCMV5 or pcDNA3.3 vector for transient transfection. HA-HIF1α-pcDNA3 (#18949) ^6^ and HA-VHL-pRc/CMV (#19999) ^7^ were ordered from Addgene (Watertown, MA, US).

**PHD2 knockout by CRISPR-Cas9**

PHD2 knockout HepG2 cells were prepared by CRISPR-Cas9–mediated gene editing. We used CRISPR design tool to select PHD2 target sequences. Potential target oligomers for sgRNA constructs were designed with a high efficiency score. PHD2 targeting gRNA was inserted into lentiCRISPR v2, and then HepG2 cells were infected with this CRISPR-Cas9 lentivirus. After infection, the cells were selected with medium containing 2.0 μg/mL puromycin.

**siRNA experiment**

Cells were cultured in the presence of negative control siRNA (Qiagen, Hilden, Germany) or PHD2 siRNA (Qiagen) for 48 h. Transfection of siRNA was performed according to the manufacturer's instructions (Qiagen). 10 μm siRNA with HiPerFect transfection reagent (Qiagen) in serum-free culture medium was incubated for 10 min at room temperature (transfection mixtures) and added directly to the cultured cells.

**Human liver tissue preparation**

All procedures involving human samples complied with the principles outlined in the Declaration of Helsinki and were approved by the Institutional Review Board of Union Hospital, Tongji Medical College, Huazhong University of Science and Technology.

Paraffin-embedded human liver tissues of patients with anemia of chronic disease (*n* = 10) and normal individuals (*n* = 10) were collected from liver biopsy samples. The main clinical data on humans are summarized in Table A. Immunohistochemistry for AMPKα1 and hydroxy-HIF-1α were performed. For immunohistochemical grading, the intensity of AMPKα1 and Hydroxy-HIF-1α were defined as 0, 1+, 2+, or 3+. The immunoscore (H score) was defined by the intensity (0–3+) multiplied by the expression percentage (0–100) for each sample.

The staining H score was calculated, and then median value was chosen as the cutoff value^8, 9^. Therefore, samples with an H score ≥ median value had high expression/level, and samples with an H score < median value had low expression/level. Slides were scored without knowledge of any clinical information, and the final staining score was the average of scores from 2 independent pathologists.

**Reference**

1. Eng JK, McCormack AL and Yates JR. An approach to correlate tandem mass spectral data of peptides with amino acid sequences in a protein database. *J Am Soc Mass Spectrom*. 1994;5:976-89.

2. Elias JE and Gygi SP. Target-decoy search strategy for increased confidence in large-scale protein identifications by mass spectrometry. *Nat Methods*. 2007;4:207-14.

3. Beausoleil SA, Villen J, Gerber SA, Rush J and Gygi SP. A probability-based approach for high-throughput protein phosphorylation analysis and site localization. *Nat Biotechnol*. 2006;24:1285-92.

4. Wang C, Dai X, Wu S, Xu W, Song P and Huang K. FUNDC1-dependent mitochondria-associated endoplasmic reticulum membranes are involved in angiogenesis and neoangiogenesis. *Nat Commun*. 2021;12:2616.

5. Bogdanova A, Mihov D, Lutz H, Saam B, Gassmann M and Vogel J. Enhanced erythro-phagocytosis in polycythemic mice overexpressing erythropoietin. *Blood*. 2007;110:762-9.

6. Kondo K, Klco J, Nakamura E, Lechpammer M and Kaelin WG, Jr. Inhibition of HIF is necessary for tumor suppression by the von Hippel-Lindau protein. *Cancer Cell*. 2002;1:237-46.

7. Iliopoulos O, Kibel A, Gray S and Kaelin WG, Jr. Tumour suppression by the human von Hippel-Lindau gene product. *Nat Med*. 1995;1:822-6.

8. Shi B, Abrams M and Sepp-Lorenzino L. Expression of asialoglycoprotein receptor 1 in human hepatocellular carcinoma. *J Histochem Cytochem*. 2013;61:901-9.

9. Liu D, Zhang XX, Li MC, Cao CH, Wan DY, Xi BX, Tan JH, Wang J, Yang ZY, Feng XX, Ye F, Chen G, Wu P, Xi L, Wang H, Zhou JF, Feng ZH, Ma D and Gao QL. C/EBPbeta enhances platinum resistance of ovarian cancer cells by reprogramming H3K79 methylation. *Nat Commun*. 2018;9:1739.

**Supplementary Figures**

**
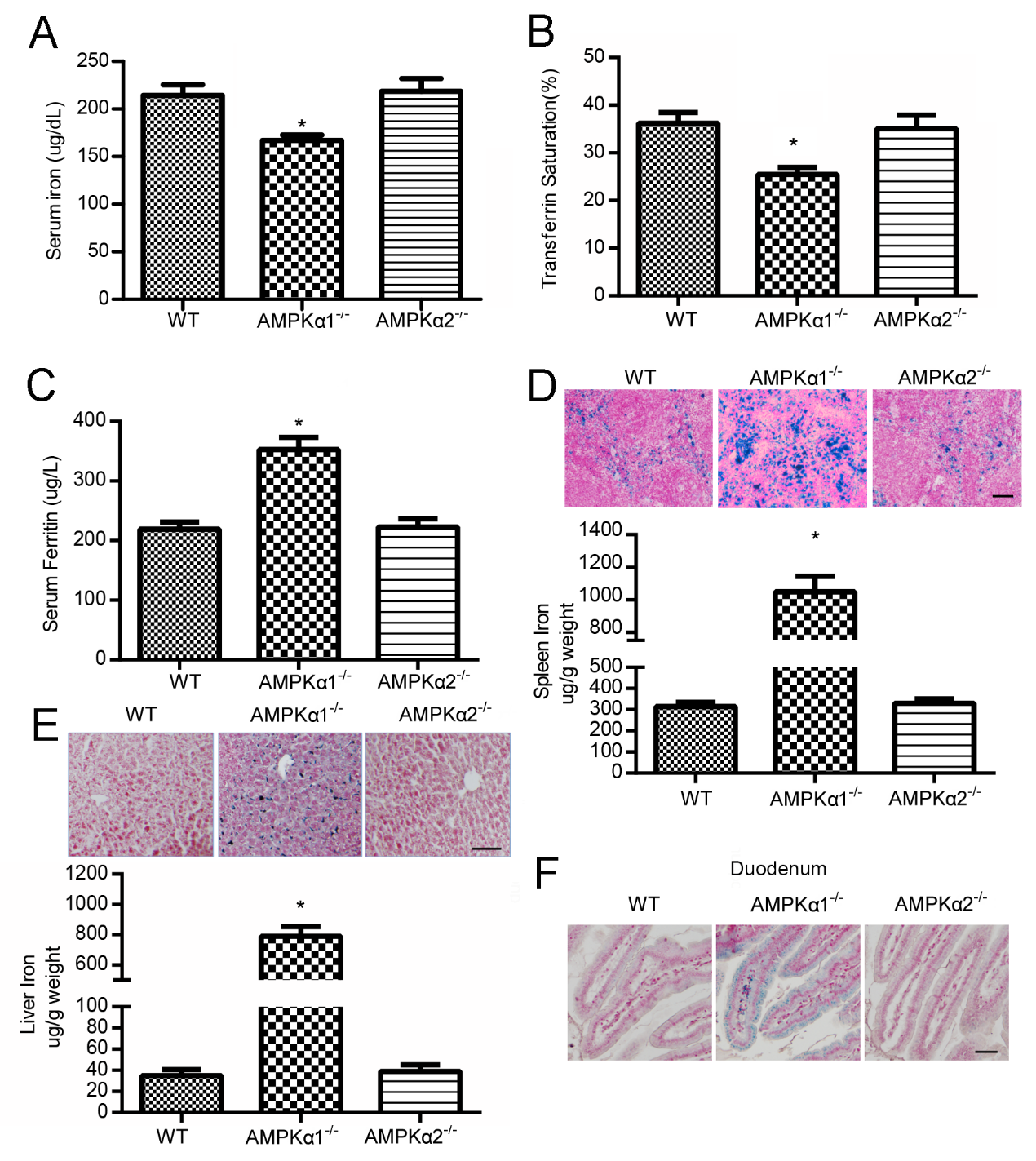
**

**Figure S1. AMPKα1^−/−^ mice display decreased levels of serum iron and increased iron deposition in the liver and duodenum.** Serum iron concentration (A) and transferrin saturation (B) in WT, AMPKα1^−/−^, and AMPKα2^−/−^ mice. **p* < 0.05 versus WT (*n* = 14). (C) Serum ferritin levels in WT, AMPKα1^−/−^, or AMPKα2^−/−^ mice. **p* < 0.05 versus WT (*n* = 10). (D–F) Perls Prussian blue staining in sections of the spleen (D), liver (E), and duodenum (F) from WT, AMPKα1^−/−^, or AMPKα2^−/−^ mice. Nonheme iron was measured by using the bathophenanthroline iron assay. Bar = 50 µm. **p* < 0.05 versus WT (*n* = 10).


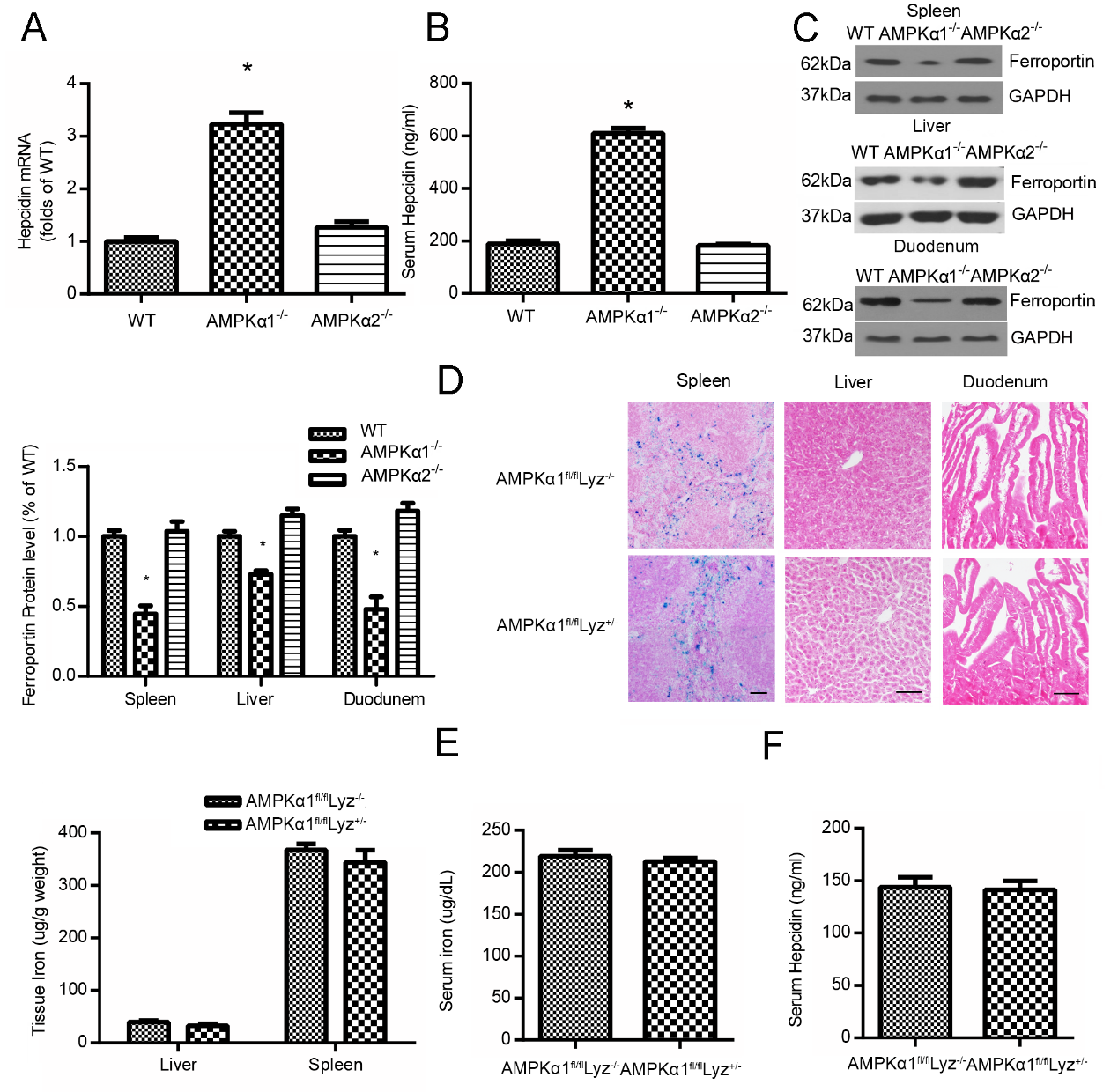


**Figure S2. High levels of hepcidin parallel low levels of ferroportin in universal AMPKα1^−/−^ mice, but not in hematopoietic-specific AMPKα1^−/−^ mice.** (A) Real-time PCR analysis of hepcidin mRNA expression in liver samples from WT, AMPKα1^−/−^, or AMPKα2^−/−^ mice. **p* < 0.05 versus WT (*n* = 10). (B) Serum hepcidin level from WT, AMPKα1^−/−^, or AMPKα2^−/−^ mice. **p* < 0.05 versus WT (*n* = 10). (C) Western blot analysis of ferroportin expression in the spleen, liver, and duodenum. GAPDH was used as the control. **p* < 0.05 versus WT (*n* = 5). (D) Perls Prussian blue staining in spleen, liver, and duodenum sections and (E) serum iron and (F) hepcidin measurements of AMPKα1^fl/fl^Lyz^+/−^ and AMPKα1^fl/fl^Lyz^−/−^ mice. **p* < 0.05 versus AMPKα1^fl/fl^Lyz^−/−^ (*n* = 10).

**
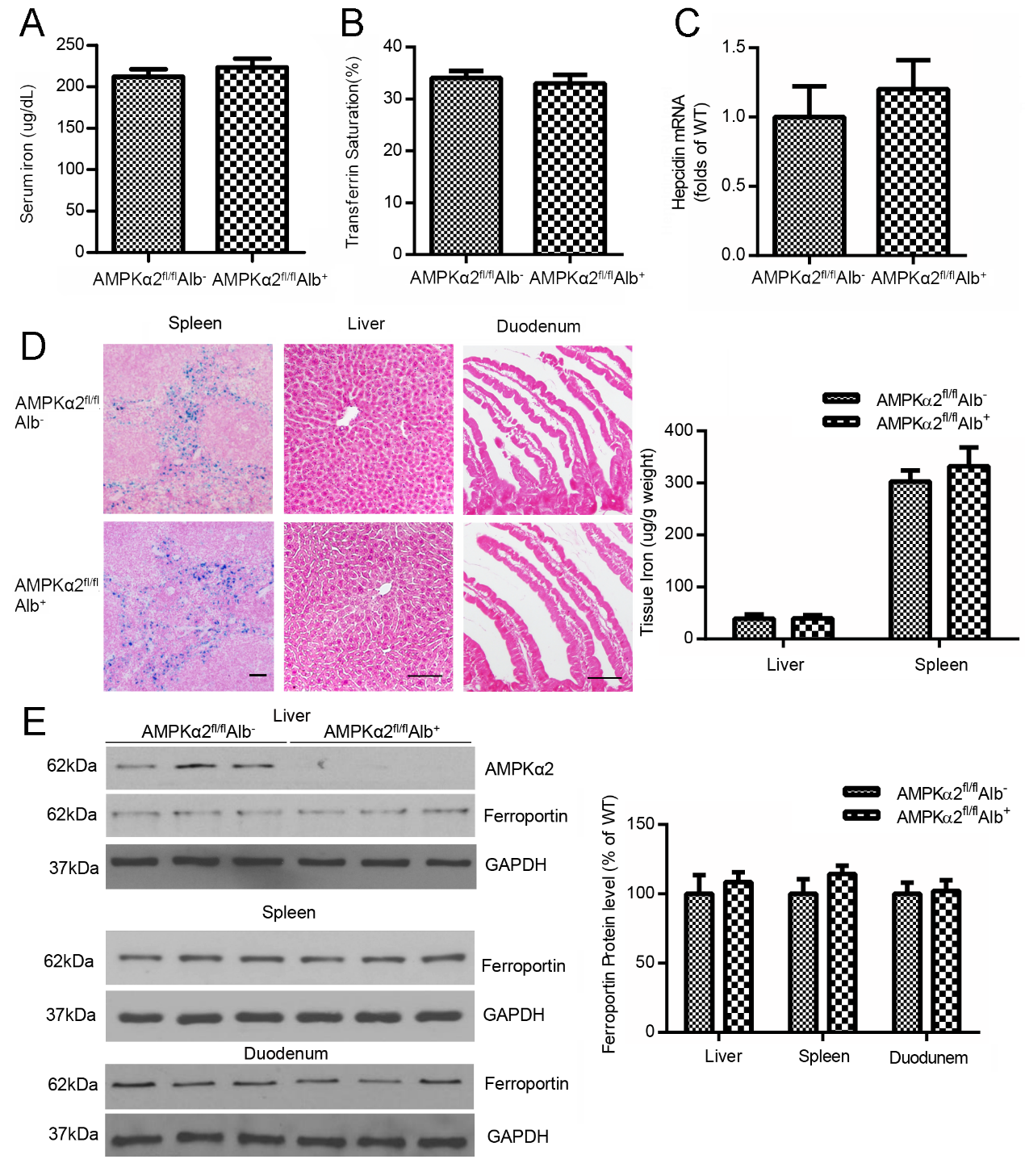
**

**Figure S3. Liver-specific AMPKα2 knockout mice did not display hypoferriemia and ion deposition.** (**A**) Serum iron concentrations and (**B**) transferrin saturation were measured in AMPKα2^fl/fl^ Alb^+^ and AMPKα2^fl/fl^ Alb^-^ mice. *p < 0.05 vs. WT (n=8). (**C**) Real-time PCR analysis of hepcidin mRNA levels in liver samples from AMPKα2^fl/fl^Alb^+^ and AMPKα2^fl/fl^ Alb^-^ mice, *p < 0.05 vs. AMPKα2^fl/fl^ Alb^-^ (n=5). (**D**) Perls Prussian blue staining was performed in sections of spleen, liver and duodenum from AMPKα2^fl/fl^Alb^+^ and AMPKα2^fl/fl^ Alb^-^ mice. Bar=50 µm. (**E**) Western blot analysis of ferroportin expression from spleen, liver and duodenum in AMPKα2^fl/fl^Alb^+^ and AMPKα2^fl/fl^ Alb^-^ mice, *p < 0.05 vs. AMPKα1^fl/fl^ Alb^-^ (n=5).


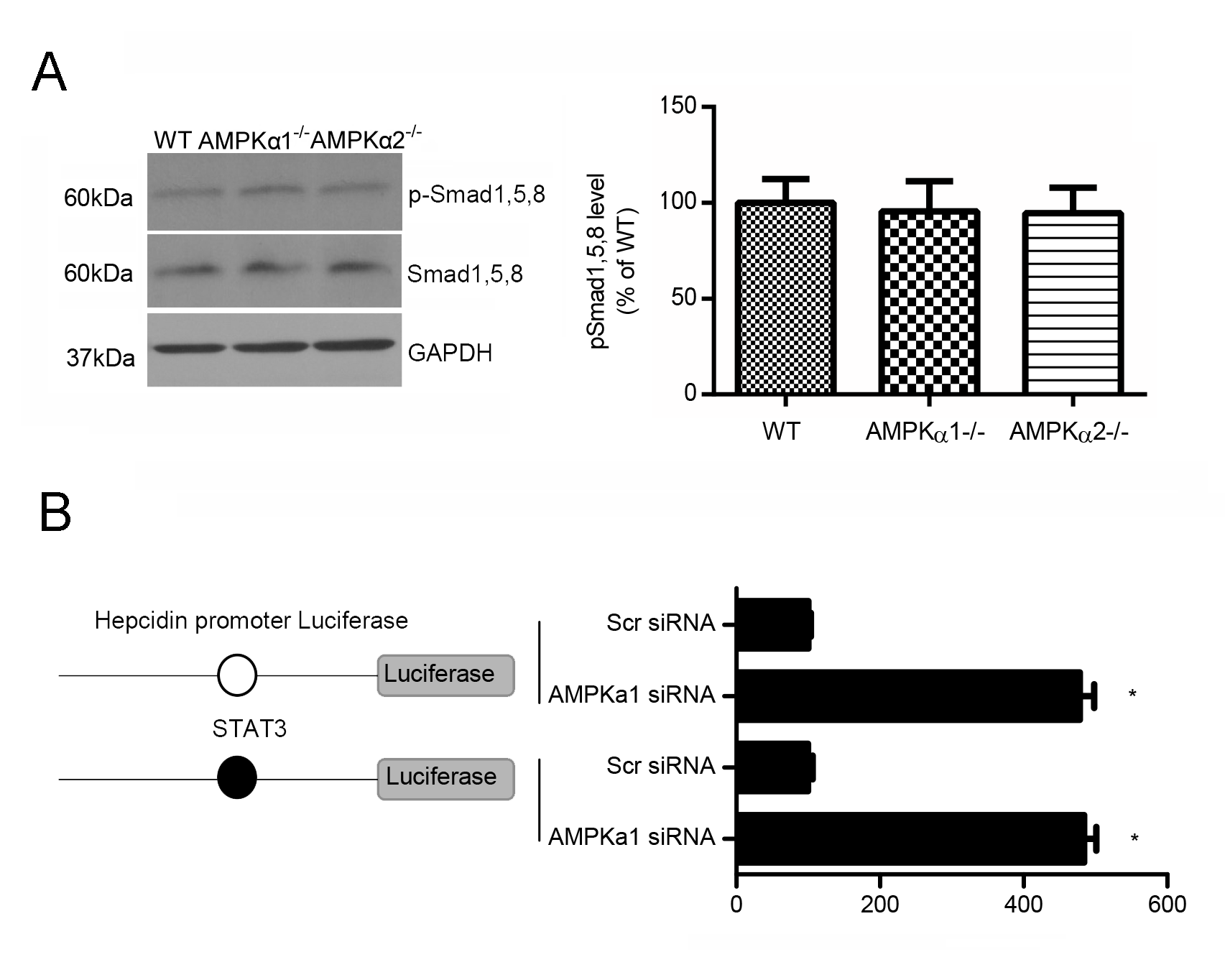


**Figure S4. The effect of AMPKα1 on hepcidin are not attributed to the SMAD and STAT3 signaling pathway.** (**A**) Western blot analysis of p-Smad1,5,8 and Smad1,5,8 expression in the livers of WT, AMPKα1^-/-^, and AMPKα2^-/-^ mice. *p < 0.05 versus WT (n = 5). (**B**) HepG2 cells were pre-transfected with Scr siRNA and AMPKα1 siRNA for 24 h, then transfected with WT HAMP or the deletion (STAT3 binding site) HAMP luciferase promoter for 24 h. After this, luciferase activity was measured. *p < 0.05 versus Scr siRNA (n = 7).


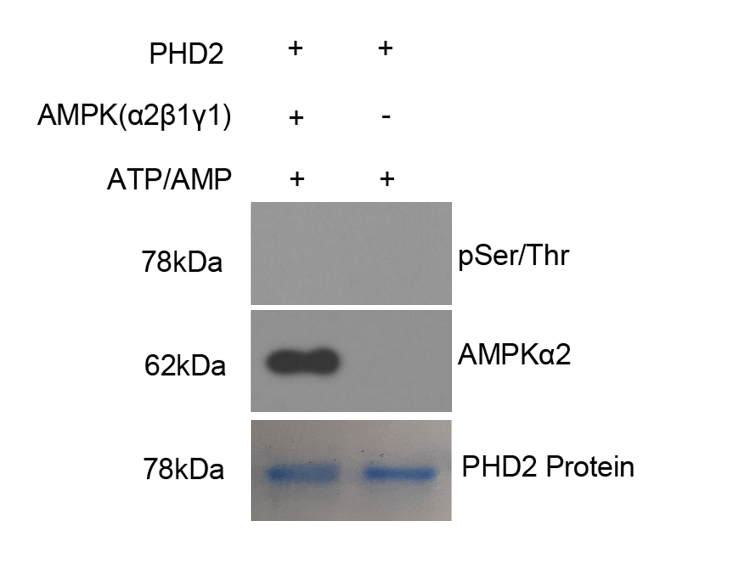


**Figure S5. AMPKα2 could not phosphorylated PHD2.** In vitro kinase assay, purified PHD2 protein was incubated with and without AMPKα2β1γ1 complex kinase and followed by western blot analysis of the samples.


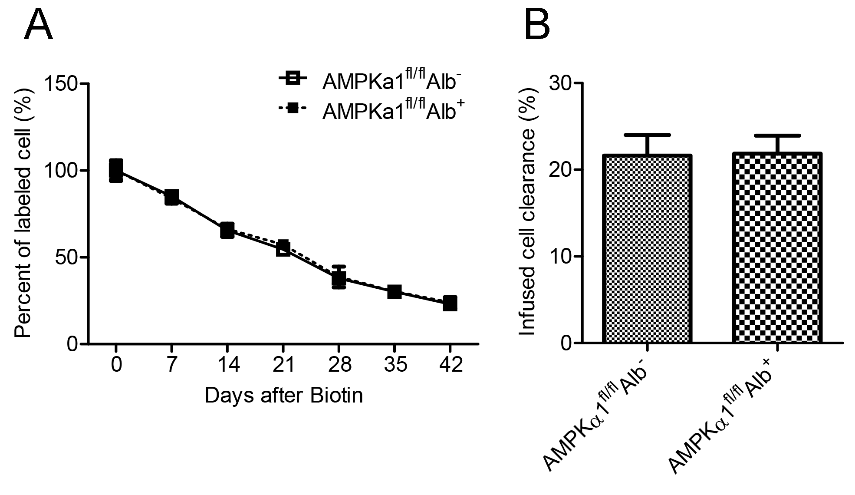


**Figure S6. Liver-specific AMPKα1 knockout mice have no defect of erythrocytes.** (**A**) Representative experiments showing decreased life span of erythrocytes in AMPKα1^fl/fl^Alb^+^ and AMPKα1^fl/fl^ Alb^-^ mice (n=5 in each group). (**B**) Clearance of reinfused biotin-labeled blood cells in AMPKα1^fl/fl^Alb^+^ and AMPKα1^fl/fl^ Alb^-^ mice (n=5 in each group).

**Table A. Clinical and biochemical characteristics of human samples**

|  | Control | Anemia of chronic Disease (ACD) |
| --- | --- | --- |
| Age (years) | 50±3.5 | 58±4.0 |
| Sex (M/F) | 7/3 | 7/3 |
| RBC (10^12^/L) | 4.65±0.15 | 3.68±0.09^*^ |
| Hgb (g/dl) | 136.2±7.5 | 89.8±2.4^*^ |
| MCV (fl) | 91.2±1.5 | 88.5±0.8 |
| MCH (pg) | 30.9±0.7 | 28.6±1.1 |
| Serum Iron (umol/L) | 21.3±1.6 | 6.9±0.6^*^ |
| Serum Ferritin (ng/ml) | 113.9±5.9 | 130.8±4.5^*^ |

The age, Hgb (Hemoglobin), serum iron and serum ferritin levels as well as gender in Control (Liver transplantation donor) or anemia of chronic disease (ACD) patients (patients who underwent liver operation). All data are expressed as means ±SEM.
